# Involvement in maternal care by migrants and ethnic minorities: a narrative review

**DOI:** 10.1186/s40985-020-00121-w

**Published:** 2020-04-07

**Authors:** Cláudia De Freitas, Janka Massag, Mariana Amorim, Sílvia Fraga

**Affiliations:** 1 EPIUnit – Instituto de Saúde Pública, Universidade do Porto, Rua das Taipas, 135, 4050-600 Porto, Portugal; 2 Departamento de Ciências da Saúde Pública e Forenses e Educação Médica, Faculdade de Medicina, Universidade do Porto, Porto, Portugal; 3 Centre for Research and Studies in Sociology, University Institute of Lisbon (ISCTE-IUL), Lisbon, Portugal

**Keywords:** User involvement, Patient participation, Maternal health services, Migrant, Ethnic minority

## Abstract

**Background:**

Guidelines for improving the quality of maternal health services emphasise women’s involvement in care. However, evidence about migrant and ethnic minorities’ preferences for participation in maternal care remains unsystematised. Understanding these populations’ experiences with and preferred forms of involvement in care provision is crucial for imbuing policies and guidelines with sensitivity to diversity and for implementing people-centred care. This paper presents a narrative synthesis of empirical studies of involvement in maternal health care by migrants and ethnic minorities based on four key dimensions: information, communication, expression of preferences and decision-making.

**Methods:**

Studies indexed in PubMed and Scopus published until December 2019 were searched. Original quantitative, qualitative and mixed methods studies written in English and reporting on migrant and ethnic minority involvement in maternal care were included. Backward reference tracking was carried out. Three researchers conducted full-text review of selected publications.

**Results:**

In total, 22 studies met the inclusion criteria. The majority of studies were comparative and addressed only one or two dimensions of involvement, with an emphasis on the information and communication dimensions. Compared to natives, migrants and ethnic minorities were more likely to (1) lack access to adequate information as a result of health care staff’s limited time, knowledge and misconceptions about women’s needs and preferences; (2) report suboptimal communication with care staff caused by language barriers and inadequate interpreting services; (3) be offered fewer opportunities to express preferences and to have preferences be taken less into account; and (4) be less involved in decisions about their care due to difficulties in understanding information, socio-cultural beliefs and previous experiences with care provision less attuned with playing an active role in decision-making and care staff detracting attitudes.

**Conclusion:**

Constraints to adequate and inclusive involvement in maternal care can hinder access to quality care and result in severe negative health outcomes for migrant and ethnic minority women. More research is needed into how to tailor the dimensions of involvement to migrant and ethnic minorities’ needs and preferences, followed by provision of the resources necessary for effective implementation (e.g. sufficient time for consultations, optimal interpreter systems, health care staff training).

## Background

The number of international migrants has been growing globally, and it is now estimated at 258 million [1]. High population mobility is unlikely to come to a halt. Millions of people are on the move driven by personal aspirations, increased opportunities for travel, but also the need to flee extreme poverty, war, persecution and the negative consequences of climate change. The rising number of people living outside their countries of origin poses a public health challenge as migrants and ethnic minorities tend to be more negatively affected by inequities in health status and access to health care than native populations [2].

Access to maternal care is reported to be worse for migrants than for natives, especially for displaced and refugee populations and those with irregular status and a low socioeconomic position [3, 4]. This is particularly problematic because pregnant women are an especially vulnerable group and limited access to needed care impends on their right to health and healthy child development [3]. Language and cultural differences, as well as institutional discrimination and structural barriers, have been reported as factors that may reduce migrant and ethnic minority women’s access to maternal health services [4, 5]. Evidence also suggests that migrant and ethnic minority women experience increased maternal mental ill-health and maternal and perinatal mortality compared to natives [6–10]. Evidence on other perinatal outcomes is mixed. While most studies have shown poorer outcomes among migrants (e.g. complicated pregnancies, low birth weight, preterm delivery, congenital malformations, abortion), some studies reported improved outcomes (e.g. pre-eclampsia, eclampsia, breastfeeding, low birth weight) and other studies have found no differences between migrant and native groups (e.g. pregnancy complications, preterm delivery) [5, 11–17].

Maternal and child health is a public health priority under the Sustainable Development Goals [18]. As a result, guidelines concerned with maternal care improvement have been published in recent years. Recommendations are often based on the premise that women want to be involved in care decision-making, and their focus is set on electing women’s preferences and choices through effective communication [19]. Service user involvement has been found to improve treatment outcomes [20], patient safety [21] and care accessibility [22, 23], which are all key elements of quality care [24]. However, evidence about migrants and ethnic minorities’ perceptions of involvement in maternal care is limited and unsystematic.

User involvement in health care is a multifaceted phenomenon “through which individuals formulate meanings and actions that reflect their desired degree of participation in individual [ ] decision-making processes” [25]. As such, it needs to be viewed as a dynamic process of co-production that is grounded in dialogue and negotiation between the parties involved (i.e. service users and care professionals) [25, 26] and which can lead to disparate outcomes regarding decision-making (from full willingness to make decisions to preferred delegation) [27]. Understanding migrants and ethnic minorities’ experiences with and preferred forms of involvement is crucial for imbuing guidelines and policies with sensitivity to diversity [28–30], implementing care centred on people’s needs, values and preferences [31–33] and avoiding the reproduction of inequalities through promotion of inadequate or undesired participation [34, 35]. This paper aims to synthesise existing knowledge about migrant and ethnic minority involvement in maternal care by providing a narrative review of empirical studies on this issue.

## Defining user involvement in health care

User involvement in health care started gaining currency in the late 1970s [36]. Emerging as a reaction to paternalistic professionalism that restricted patients’ agency over their own health and care management [37], it was rebranded as a patient choice in the 1980s, following the rise of New Public Management and its precept of cost containment through the promotion of patients’ autonomy and responsibilisation (e.g. adoption of healthy lifestyles) [38]. Towards the end of the twentieth century, user involvement regained its original dialogic aura under the philosophy of people-centred care [39]. At present, it is a core dimension in guidelines for care quality improvement, not least in the field of maternal care [40, 41] where access to quality care is promoted as a right [4].

Despite increasing recognition and practice, there is no consensual definition of user involvement. Terms such as “involvement”, “participation” and “engagement” are used interchangeably, though not always with the same meaning [26, 42, 43]. Definitions of user involvement tend to differ on the emphasis given to decision-making and to the roles awarded to the actors involved, i.e. service users and health professionals. Some authors focus on the dialogic relationship underlining user involvement. They assert the need for four basic elements to be present for involvement to unfold: (a) a respectful relationship between health professionals and service users, (b) commitment to reduce the knowledge gap between the parties involved through the provision of adequate information, (c) devolution of power to service users by health professionals and (d) opportunity for involvement in treatment decision-making by users to the extent they see fit [37, 44–46]. Other authors emphasise the act of decision-making and health professionals’ leading role in involving service users by providing information, clarifying doubts, actively inviting users to participate and taking their opinions and wishes into account [47–49].

The centrality of decision-making on the process of user involvement has been criticised on several accounts. Studies show that even when service users are unwilling to participate in decision-making, they still value being involved, namely by receiving information from health professionals and having them take their preferences into account [27, 50]. Research further shows that service users want to be involved in decision-making to different degrees [51–53] and that, when involved at the preferred level, they experience positive health outcomes as a result [54]. Understanding what involvement means for service users, and the subjective feelings it invokes on both users and professionals, has thus been advocated as a key stepping stone in attempts geared towards its definition [55].

Andrew Thompson (2007) studied lay people’s views of and preferences for involvement in health care following a deliberative design that included a group of participants differing in age, gender, ethnicity, social class, health needs and experiences with the health care system. According to the study participants, involvement entailed one or more of the following attributes: information, explanation, openness, communication, shared knowledge, emotional care, exploration of choices, dialogue and decision-making. Based on the most frequently mentioned attributes, Thompson proposes an empirically grounded definition of user involvement that entails a range of steps where participation in decision-making is presented as an opportunity, rather than an obligation. User involvement can thus be “broadly understood as involving patients in discussion about their condition, providing them with relevant information, asking for their opinion on possible treatments, and involving them in the decision-making process, should they so wish” [56]. This definition highlights four core dimensions of involvement: information, communication, expression of preferences and decision-making. These dimensions are used to organise our review of migrant and ethnic minority involvement in maternal care.

## Methodology

A narrative review was conducted to identify empirical research about migrant and ethnic minority involvement in maternal care. Relevant references were retrieved by searching the electronic databases PubMed and Scopus in December 2019. The search expression combined sets of terms relating to user involvement (involvement, participation, engagement), maternal care (maternal, reproductive, perinatal, antenatal) and migrant and minority populations (migrant, immigrant, ethnic minority, asylum seeker, refugee). Only original full-length empirical studies written in English were considered (reviews, editorials and commentaries were excluded). All relevant quantitative, qualitative and mixed methods studies reporting data on involvement in maternal care by migrant and ethnic minorities were included, independently of addressing only one or more of the four dimensions of involvement described earlier. Backward reference tracking of the articles included was also carried out.

In total, 91 references were generated and 22 publications met the inclusion criteria. Full-text review of selected publications was independently conducted by three researchers. Data extraction from each publication was carried out and tabulated (Table 1). The main themes for analysis were deductively drawn from Thompson’s definition of user involvement [56] selected for the purposes of this review, and categories were established inductively through analysis of the publications.

**Table 1:** Empirical studies examining migrant and ethnic minority involvement in maternal care (*n* = 22)

Authors	Year of publication	Country of study	Study approach and design	Study populations	Sample size	Main findings
Almeida and Caldas	2013	Portugal	Qualitative approach	Recent mothers, with children under 36 months, whose parents were not born in Portugal (Brazilian immigrants) and Portuguese nationals, residing in Porto Metropolitan Area	14 participants (7 Portuguese women; 7 Brazilian women)	Brazilian women expressed dissatisfaction with the following: quality of information provided by health professionals and their communication skills, insufficient duration of appointments, bureaucracy in primary care, limited access to specialised care and insufficient preventive care. Misinformation about legal rights and inappropriate clarification in medical appointments interacted with social determinants and resulted in poorer medical care.
Almeida et al.	2014a	Portugal	Qualitative approach	Recent mothers, with children under 36 months, immigrants and Portuguese natives, living in Porto Metropolitan Area	31 participants (6 Portuguese women; 11 women born in Portuguese-speaking African countries; 7 women born in Eastern European countries; 7 women born in Brazil)	During postpartum, women (irrespective of nationality) reported a significant lack of social and affective medical support. Immigrants reported difficulties in understanding professionals during medical consultations with their infants. Eastern European women who showed interest in engaging in shared decision-making regarding treatment reported that doctors frequently seemed unprepared to answer questions and to find it difficult to obtain information about the NHS. African women revealed language barriers and lack of active communication with professionals. Brazilian and Eastern European women reported dissatisfaction with baby follow-up in primary care.
Almeida et al.	2014b	Portugal	Qualitative approach	Recent mothers, with children under 36 months, immigrants and Portuguese natives, living in Porto Metropolitan Area	31 participants (6 Portuguese women; 11 women born in Portuguese-speaking African countries; 7 women born in Eastern European countries; 7 women born in Brazil)	Brazilian and Eastern European women reported strictness by health care providers, which often triggered their inhibition in asking questions to clarify issues and returning when facing complications. Eastern European women reported that doctors were unprepared to answer questions and felt uncomfortable discussing health procedures with well-informed patients. They also reported language barriers to hinder their understanding of clinical procedures. Misinformation and inadequate clarification during medical appointments resulted in perceived lower quality care.
Ascoli et al.	2001	Netherlands	Qualitative approach	Refugee women from different countries of origin, with varying status within the asylum-seeking system and different living situations (single or married, urban or rural), and health care providers (doctors, nurses, midwives and others) who deal with pregnant women in their work	Not reported: 4 refugee women (1 Guinean, 1 Afghan, 1 Somali and 1 Iranian); several health care workers	The experience of reproductive care by refugee women is influenced by their status and by their gender. Refugee women revealed informational needs, communication concerns and serious financial and legal issues that shape their pregnancy and delivery experiences. Weak points in the health care system: limited time for consultations and obstacles to accessibility.
Attanasio et al.	2018	USA	Quantitative approach; prospective cohort study	Women who gave birth, vaginally or by unplanned caesarean, to a first, singleton baby in a Pennsylvania hospital between 2009 and 2011	2787 participants (2325 White women; 200 Black women; 150 Latin women; 112 women with other or multiple race)	Black women and those who did not have a college degree or private insurance or who underwent labour induction, instrumental vaginal or caesarean delivery were less likely to report high shared decision-making. Disproportionately less engaged decision experience for pregnant patients in more marginalised social groups.
Binder et al.	2012	England	Qualitative approach	Somali and Ghanaian immigrant women and white British women who had at least one child within the British health care system and who were living in Greater London between 2005 and 2006; 62 obstetric care providers (doctor and midwives) at five hospitals within the study area	122 participants (10 white British women; 39 immigrant Somali women; 11 immigrant Ghanaian women; 62 obstetric care providers)	Women and providers encountered difficulties in communication; language was the main barrier, especially for Somalis. Professionalism and competence were considered more important than meeting providers from one’s own ethnic group. All groups acknowledge the importance of the interpreter’s role in health communication.
Davies and Bath	2001	UK	Qualitative approach	Somali women living in a Northern English city who had used maternity and women’s health services in that city	13 Somali women	Women experienced difficulties in identifying sources of information other than their GP. Poor communication with health workers was a problem in seeking information for non-English-speaking women. Fears about misinterpretation and confidentiality limit the usefulness of interpreters. Somali women perceived that they were denied information due to punitive attitudes and prejudiced views among health professionals.
Esscher et al.	2014	Sweden	Quantitative approach; retrospective data analysis	Maternal death data records among foreign-born women from low- and middle-income countries and Swedish-born women collected from Swedish official and national registries for 1988 to 2007 and from the Swedish Society of Obstetrics and Gynaecology Maternal Mortality Group for 2008 to 2010	75 maternal death records	Major and minor suboptimal factors were associated with a majority of maternal deaths, more often to foreign-born women. The main delays to care-seeking were non-compliance among foreign-born women and communication barriers, such as incongruent language and suboptimal interpreter system or usage. Inadequate care occurred more often among the foreign-born.
Henderson et al.	2013	England	Quantitative approach; cross-sectional study	Women aged 16 years and over, living in England in 2010, who had recently given a live birth	24,319 women (20,633 self-identified as White (84.8%); 3686 self-identified as coming from seven ethnic groups: Mixed (1.3%), Indian (2.4%), Pakistani (2.5%), Bangladeshi (0.7%), Black Caribbean (0.7%), Black African (2.7%), other (5.0%))	Women from minority groups access antenatal care later in pregnancy, have fewer antenatal checks, fewer ultrasound scans and less screening. They were less likely to receive pain relief in labour. They had longer lengths of hospital stay and were more likely to breastfeed, but they had fewer home visits from midwives. Throughout maternity care, they were less likely to feel spoken to so they could understand, to be treated with kindness, to be sufficiently involved in decisions and to trust in health staff.
Henderson et al.	2018	England	Quantitative approach; cross-sectional national survey	Women aged 16 years or over who delivered a live single birth in October or November 2009	5235 women (4108 women born in the UK; 194 born in Accession countries; 169 born in the old European Economic Area; 764 born in the rest of the world)	Migrants reported a poorer experience of care than UK-born women. Recent migrants from the Accession countries were significantly less likely to feel that they were spoken to so they could understand and treated with kindness and respect.
Hennegan et al.	2014	Australia	Quantitative approach; cross-sectional population-based survey	Women who had a live singleton or multiple birth in Queensland, Australia, between February and May 2010	6050 participants (5569 Australian-born English-speaking women; 481 women born in another country who spoke a language other than English at home)	Most women felt that they were treated as an individual and with kindness and respect. Women born outside Australia were less likely to report being looked after “very well” during labour and birth and to be more critical of care.
Hennegan et al.	2015	Australia	Quantitative approach; cross-sectional population-based survey	Women who had a live singleton or multiple birth in Queensland, Australia, between February and May 2010	2955 participants (2722 Australian-born English-speaking women; 233 women born in another country who spoke a language other than English at home)	Women born outside Australia were less likely to report pain after birth was manageable, or rate overall postnatal physical health positively. They more frequently reported having painful stitches, distressing flashbacks and feeling depressed; they were less likely to feel involved in decisions and to understand their options for care; they were more likely to report being visited by a care provider at home after birth.
Higginbottom et al.	2016	Canada	Qualitative approach; ethnographic study	Immigrant women with current or recent (previous 2 years) experience of using maternity services; health care providers having experience with providing perinatal care to immigrant women; stakeholders who have a mandate or involvement in immigrant women’s health or service provision, including social service providers and decision-makers	86 participants (34 immigrant women; 29 health care providers; 23 social services providers [including some other key stakeholders])	Barriers to access and navigation of maternity services by immigrant women in Canada: communication difficulties, lack of information, lack of social support (isolation), cultural beliefs, inadequate health care services and cost of medicine/services. Immigrant women face additional challenges that influence their level of satisfaction and quality of care: lack of understanding of the informed consent process, lack of regard by professionals for confidential patient information, short consultation times, short hospital stays, perceived discrimination/stereotyping and culture shock.
Jonkers et al.	2011	Netherlands	Qualitative approach	Immigrant and native Dutch women with severe maternal morbidity	50 women (10 native Dutch women; 40 immigrants)	Immigrant women reported that health care providers paid insufficient attention to their pregnancy-related complaints; delays in receiving information about diagnosis and treatment; problems identifying medically complications, presenting their complaints to health providers effectively and taking an active role as patients. Highly educated migrants showed low health literacy skills in interaction with doctors.
Lindsay et al.	2016	USA	Qualitative approach	Brazilian-born immigrant women residing in two cities in the greater Boston area (Somerville and Brighton), USA	35 Brazilian-born immigrant women	Participants expressed overall satisfaction with the US health care system. Barriers to care: sociocultural differences in care delivery and communication barriers, including inconsistent quality of interpreting services.
Philibert et al.	2008	France	Quantitative approach; case-control study	Women who died of maternal death from 1996 to 2001 as cases and a representative sample of women who gave birth in 1998 as controls	13,453 women (267 women who died during the postpartum period as case subjects [20.6% non-French women]; 13,186 women as control subjects [10.6% non-French women])	The risk of postpartum maternal death was twice as high for foreign women. The risk of dying from hypertensive disorder or infection was four times higher for foreign women. Among women who died, care was more often considered not optimal for foreign women.
Phillimore	2016	UK	Mixed methods approach	Women who moved to the UK within the past 5 years and utilised maternity services; maternity professionals	100 participants (82 migrant women; 18 individuals working regularly with migrant women)	New migrant women were more likely to book late or fail to attend follow-ups than the general population. A combination of structural, legal and institutional barriers prevents migrant women accessing effective antenatal care.
Redshaw and Heikkila	2010	England	Quantitative approach; cross-sectional study	Women aged 16 years and over who had their baby in England within a 2-week period in October to November 2009	5333 women (21% born outside the UK; 14% from Black and Minority Ethnic groups)	Women from Black and Minority Ethnic groups experienced poorer staff communication and feelings about not being treated with respect. Single women, those who had left education at 16 years or earlier, living in the most deprived areas and Black and Minority Ethnic women were less likely to have seen a health professional by 12 weeks about their pregnancy care or to be aware of all the options for where they could give birth.
Redshaw and Henderson	2015	England	Quantitative approach; cross-sectional study	Women aged 16 years and over who had their baby in England within a 2-week period at the beginning of January 2014	4571 women (24% born outside the UK; 16% from Black and Minority Ethnic groups)	Women from Black and Minority Ethnic groups and women born outside the UK were later in accessing care. Women from Black and Minority Ethnic groups experienced poorer staff communication and feelings about not being treated with respect. Single women were more likely to access care later, less likely to feel involved in decisions about their care and more likely to feel left alone and worried during their care and were less satisfied overall. Women living in the most disadvantaged areas were more likely to feel involved in decisions about their antenatal care and less likely to see a midwife after the baby had reached 2 weeks of age.
Redshaw et al.	2007	England	Quantitative approach; cross-sectional study	Women aged 16 years and over who had their baby in England in 2 specific weeks: 2–8 January and 4–10 March 2006	3198 women (17% born outside the UK; 13% from Black and Minority Ethnic groups)	Black and Minority Ethnic women, those born outside UK, women living in deprived areas and women who are single parents were more likely to recognise their pregnancy later, to first see a health professional later and to book later for antenatal care. They were less likely to have felt that they were treated with respect and talked to in a way that they could understand by staff during pregnancy, labour and birth and postnatal care.
Reitmanova and Gustafson	2008	Canada	Qualitative approach	Immigrant Muslim women aged 25–40 years who delivered at least one child in St. John’s, Canada, between 1995 and 2005	6 immigrant Muslim women	Muslim women experienced discrimination, insensitivity and lack of knowledge about their religious and cultural practices. Health information was limited or lacked the cultural and religious specificity to meet their needs during pregnancy, labour and delivery and postpartum. There were gaps between existing services and women’s needs for emotional support and culturally and linguistically appropriate information.
Yelland et al.	2015	Australia	Quantitative approach; cross-sectional consecutive population-based studies	Women giving a live birth in Victoria, Australia, in 2 weeks in 1999 and 4 weeks in 2007	4516 participant women (3578 Australian-born women; 303 overseas-born women of English-speaking background; 563 overseas-born women of non-English-speaking background)	Immigrant women of non-English-speaking background were more likely to report negative experiences of antenatal, intrapartum and postnatal care and to say that health professionals did not always remember them between visits, make an effort to get to know the issues that were important to them, keep them informed or take their wishes into account.

## Migrant and ethnic minority involvement in maternal care

Empirical evidence about involvement in maternal care by migrants and ethnic minorities is scarce, particularly regarding the expression of preferences and participation in decision-making. The majority of the studies reviewed addressed only one or two dimensions of involvement (*n* = 17), with a higher number of studies focusing on information and communication. Five studies approached three dimensions of involvement [57–61], and none addressed all of them.

Evidence derives mainly from studies conducted in the UK (*n* = 8), Australia (*n* = 3) and Portugal (*n* = 3) and published between 2001 [62, 63] and 2018 [64, 65], with an increase of research in recent years. A total of 14 out of the 22 studies reviewed were published between 2013 and 2018. An almost even number of studies adopted a quantitative approach (*n* = 11) and a qualitative approach (*n* = 10), with only one study employing a mixed methods approach. Most quantitative studies used a cross-sectional design (*n* = 9) and compared between native and migrant and ethnic minority populations (*n* = 11), but samples of the latter group were small (ranging between 8% [66, 67] and 24% [57]). The qualitative studies explored the perspectives of migrant and ethnic minority women, with four comparing between natives and migrants and three including other stakeholders (e.g. health care workers, social services providers). The mixed methods study [68] relied on questionnaires and in-depth interviews with migrant women and maternity professionals.

### Information

Access to adequate information about maternal care is not equally distributed among native and migrant and ethnic minority service users. Two population-based studies carried out in England show that Black and Minority Ethnic women born in and outside the United Kingdom (UK) were provided less information than White UK-born women [57, 61, 69]. Another population-based study on maternal care done in Australia showed that migrant women were less likely to understand the staff [58, 67] and to be kept informed during labour and birth [58] when compared to native Australian women.

Although migrants and ethnic minorities may require tailor-made information to navigate maternal care services, their informational needs are often disregarded by health professionals who either show indifference [70] or assume they have sufficient knowledge [63, 68] and use medical jargon that can increase their difficulties in understanding and realising such basic rights as informed consent [60]. This is further exacerbated by the lack of information provision regarding labour as reported by Muslim migrant women in Australia [71] and non-English-speaking Somali women in the UK [62]. The latter perceived racially prejudiced views espoused by health professionals as the main reason for not receiving enough information [62]. Health professionals’ limited time, knowledge and support to develop an understanding of the difficulties experienced by migrant women may lead to the reification of misconceptions about their behaviour (e.g. explaining failed appointments with migrant women’s undervaluing of antenatal care), which in turn may hinder the quality of clinical interactions and information sharing [68].

### Communication

Communication problems are more frequently reported by migrant and ethnic minority women when compared to natives and result in more severe consequences for the former groups. Two population-based studies carried out in England found Black and Minority Ethnic women to be less likely to be spoken to in a way that they could understand [59] and to rate communication with maternal care staff worse than native women [61]. Suboptimal communication was also more often reported by migrant women than by non-migrant women in studies undertaken in France, Sweden and England [64, 72, 73].

Limited command of the host country’s language impacts communication negatively. A study from the UK found that non-English-speaking Somali women experienced poorer communication with health professionals when compared to English-speaking Somalis [62]. Immigrant women with low proficiency in the host country’s language living in Canada, the USA, England and Portugal also experienced barriers in communication, which limited their access to maternal care services [60, 71, 74–77].

Although language barriers may be overcome through the use of interpreters, resource shortage often leads to reliance on family members, friends or children as interpreters. Suboptimal interpreter system or usage was found to be problematic and disempowering for migrant women. It caused women to be excluded from decisions regarding their pregnancies—undertaken instead by other family members [68], to fear misinterpretation and breaches in patient confidentiality [62] and to be exposed to adverse obstetric outcomes [63, 73]. Resorting to children as interpreters may also cause them extreme discomfort when they have to translate and convey bad news [74].

Poor communication was found to be associated with mortality and severe maternal morbidity among migrant women. In Sweden, suboptimal care was found to be a factor for maternal death more frequently among foreign-born women than among natives. Many of the deaths of foreign-born women were associated with communication-related barriers and delayed health care seeking (e.g. inability to access services was caused mainly by language barriers and substandard interpretation services) [73]. In the Netherlands, care providers not listening to service users and users not being able to play an active role in consultations were reported as factors for disease development among migrants but not among natives [78].

### Expression of preferences

Migrant and ethnic minority women’s preferences may be shaped by perspectives of and expectations from maternal care provision that differ from those prevailing in host countries. Those preferences may not be accommodated by local health care systems. A study carried out in the USA found that Brazilian women prefer to have access to labour ward admission and pain management procedures earlier than they are typically offered by local maternity care [77]. Inability to exercise those preferences caused Brazilian immigrants to perceive delayed admission during labour and delayed use of anaesthetics, which impacted negatively on their satisfaction with the care received [77].

Some migrant and ethnic minority groups may also be offered less opportunities to express their preferences and to see their preferences be less taken into account. A study done in the UK found that Pakistani and Black African women experienced less choice regarding the place of birth and felt significantly less likely to be able to move around during labour when compared to White, Mixed, Indian and Black Caribbean women [59]. The same study also found that Pakistani and Bangladeshi women were less likely than the other groups to feel their partners were made welcome. Two studies done in England found that Black and Minority Ethnic women born outside the UK were less aware of all options for the place of birth than UK-born White women [57, 61]. Another population-based study conducted in Australia found that immigrant women were less likely to report that care providers explained options regarding labour management than Australian-born women [58].

### Decision-making

Involvement in decision-making regarding maternal care appears to be influenced by migrant and ethnic minority women’s socio-cultural background and beliefs about and previous experiences with care provision, difficulties in accessing information and expressing preferences, health care professionals’ attitudes towards involvement and use of obstetric procedures. While some migrant groups are more acquainted with paternalistic doctor-patient relationships and expect health professionals to make decisions without involving them [74, 75], others lack the language skills to understand available options and to take part in decision-making [60, 74]. However, involvement in decision-making may also be detracted by health care professionals. A study carried out in Portugal found that although Eastern European immigrants wanted to take part in care decision-making, they felt their intents were abridged by doctors who seemed uncomfortable in answering their questions and in providing information or discussing clinical procedures with them [75, 76].

Several studies show that migrant and ethnic minority women tend to participate less in maternal care decision-making when compared to native women. Two studies carried out in England found that ethnic minority groups were less likely to report being sufficiently involved in decisions regarding antenatal care [57, 59] and during labour and birth [59] than White women. In part, this appears to be explained by limited awareness of opportunities for participation. A population-based survey done in Australia found that women born outside Australia who spoke a language other than English at home were less likely to know that they could be involved in decisions about themselves and their babies, when compared to Australian-born English-speaking women [66]. They were also less likely to be informed about all options when in need of making a decision [66].

Findings regarding respect for women’s preferences once they get involved in decision-making are inconsistent. A population survey carried out in Australia in 2008 showed that immigrant women of non-English-speaking background were more likely than native women to report that intrapartum care staff did not take their wishes into account [58]. However, another population-based survey carried out 2 years later in the same country found no differences in regard to staff’s respect for decisions made during labour by women born outside Australia who spoke a language other than English at home and Australian-born English-speaking women [67]. And yet another study conducted in the USA found that women reporting lower levels of shared decision-making during birth were disproportionately likely to be from ethnic minority groups, to be less educated and to lack health insurance [65]. The same study also found that obstetric procedures such as labour induction, assisted vaginal delivery and caesarean delivery were all associated with lower perceived involvement and that Black women who delivered by caesarean reported considerably lower levels of shared decision-making compared to White women [65].

## Conclusion

The majority of the comparative studies reviewed showed that migrants and ethnic minorities report lower levels of involvement than natives in all four core dimensions of involvement. Migrants and ethnic minorities were more likely to (1) lack access to adequate information as a result of health care staff’s limited time, knowledge and misconceptions about women’s needs and preferences; (2) report suboptimal communication with care staff caused by language barriers and inadequate interpreting services; (3) be offered fewer opportunities to express preferences and to have preferences be taken less into account; and (4) be less involved in decisions about their care due to difficulties in understanding information, socio-cultural beliefs and previous experiences with care provision less attuned with playing an active role in decision-making and care staff detracting attitudes.

Constraints to adequate and inclusive involvement in maternal care can hinder access to quality care and result in severe negative health outcomes for migrant and ethnic minority women and their offspring [73, 78]. Acting to improve their involvement in maternal care demands a multi-level approach. On the one hand, more research is needed into how to tailor the various dimensions of involvement to migrant and ethnic minorities’ needs and preferences. Further inquiry into health care staff’s beliefs, expectations and attitudes towards involvement is also needed, including how these may be influenced not only by (lack of) cultural competence but also by factors such as migrants’ origin, status and duration of stay in the host country [75, 79]. On the other hand, it is necessary to make resources available for effective implementation. This includes devising diversity sensitive information materials aimed at increasing women’s awareness of opportunities for involvement, allocating sufficient time for consultations, making optimal interpreter services accessible and training care staff to attend to the involvement needs and preferences of increasingly diverse service users. Finally, health advocacy is required to challenge systemic barriers, reduce implementation gaps and ensure that policy finds its way into practices that respect every person’s reproductive rights [29, 80, 81]. This will entail acting beyond the health sector to change discriminatory social norms and gender biases, develop inclusive policies and enforce new laws to uphold human rights. Partnerships involving multiple stakeholders working from across sectors, and at local, national and international governance levels, are key to promoting sustainable social and policy transformation [4, 82].

## Data Availability

All data generated or analysed for the purposes of the study presented are included in this article.
